# Budd-Chiari Syndrome in China: A Systematic Analysis of Epidemiological Features Based on the Chinese Literature Survey

**DOI:** 10.1155/2015/738548

**Published:** 2015-10-04

**Authors:** Wei Zhang, Xun Qi, Xitong Zhang, Hongying Su, Hongshan Zhong, Jingpu Shi, Ke Xu

**Affiliations:** ^1^Department of Radiology, The First Affiliated Hospital of China Medical University, 155 Nanjing Bei Street, Shenyang 110001, China; ^2^Key Laboratory of Diagnostic Imaging and Interventional Radiology of Liaoning Province, The First Affiliated Hospital of China Medical University, Shenyang 110001, China; ^3^Department of Clinical Epidemiology, The First Affiliated Hospital of China Medical University, Shenyang 110001, China

## Abstract

*Background.* Thousands of Budd-Chiari syndrome (BCS) studies have been published in China, and yet no one has explored its incidence or prevalence in the whole country. *Methods.* Three most commonly used Chinese language electronic databases were searched, and epidemiological data were extracted from the selected articles. *Results.* By the end of 2013, 20191 BCS cases were reported in China. The mean age of BCS patients was 36.29 ± 1.28 years, and ratio of male to female was 150/100. About 80% BCS patients were distributed in Henan, Shandong, Beijing, Jiangsu, and Anhui, and all of them except for Beijing were located in the downstream areas of Yellow River and the whole Huai River basin. The incidence and prevalence of BCS in China with and without the top 5 high-prevalence areas were estimated to be 0.88/million per year and 7.69/million and 0.28/million per year and 2.21/million, respectively. *Conclusions.* Most BCS patients in China are distributed in the downstream areas of Yellow River and the whole Huai River basin. The incidence and prevalence are comparable to those of Western countries without taking into account the top 5 high-prevalence areas.

## 1. Introduction

Budd-Chiari syndrome (BCS) was initially described as a symptomatic obstruction of the hepatic veins and subsequently broadened the definition as “hepatic venous outflow tract obstruction” which included the obstructive lesions of the suprahepatic portion of inferior vena cava (IVC) [1, 2]. In Western countries, hepatic veins thrombosis caused by thrombophilia and myeloproliferative disorders are more common than membranous obstruction of the IVC which constitutes the most cases of hepatic outflow block in developing countries especially in Asia [3–7]. Furthermore, the etiology of IVC membranous obstruction has been regarded as idiopathic [8–12], although there are evidences suggesting that the membranous obstruction of the IVC is a thrombotic sequel [13, 14]. However, there is still one key problem needed to be answered: how the thrombus initially formed without any factor found in the thrombotic risk tests.

Patients of IVC membranous obstruction are heterogeneously distributed in the glob and particularly prevail in specific counties, such as South Africa, Nepal, India, and China [5–7, 15]. This phenomenon suggests that the etiology of these patients may correlate with special epidemiological factors. However, due to the rarity of BCS, there are few nationwide epidemiological investigation sets of data reported except for the patients from Japan, France, and Sweden [16–18]. In China, a local epidemiological investigation of BCS was carried out in a country in Shandong province by Wang and his colleagues in 1988 [6], and the prevalence was estimated to be 6.4/100,000 which was obviously higher than 2/1,000,000 estimated in Western countries [19]. Although some studies have reported hundreds even thousands of BCS cases in China, which demonstrates that patients with BCS are not rare [6, 20–23], there is no evaluation of the incidence and prevalence in the whole country. Considering the huge number of Chinese population, a nationwide epidemiological investigation will be a great challenge with enormous expense of human efforts and financial resources. Is there any alternative way to do the epidemiological investigation?

Fortunately, thousands of Chinese language articles published on BCS from almost every province of China make it possible to perform a literature survey on the collection of epidemiological data. The aim of our study was to reveal the epidemiological features of BCS in China based on a literature survey.

## 2. Methods

### 2.1. Search Procedure

Three most commonly used Chinese language electronic databases, including the China National Knowledge Infrastructure (CNKI) database, the Chinese Scientific and Technological Journal (VIP) database, and the Wanfang database, were searched in March 2014. Articles searched on “Budd-Chiari syndrome” were published before 2014, and the type of publication was limited to journal articles. Considering the item “Budd-Chiari syndrome” could be translated into different Chinese characters with the same or similar pronunciation, we searched the standard translated name (Bu Jia Zong He Zheng) first and then summarized the possibly associated translated names (i.e., Bu Ka Zong He Zheng, Bai Cha Zong He Zheng, Ba De-Ji Ya Li Zong He Zheng, etc.) from the references. For the CNKI database, the translated names of Budd-Chiari syndrome (standard translated name and possibly associated translated names) were searched under the tag term of “Subject,” while, in the other two databases, the searches were done under the tag term of “Title or Keywords.” The author W. Zhang carried out all search procedures and obtained full texts. For some articles which were not correctly recorded in the database, W. Zhang connected the technical service personnel via email in order to obtain the correct articles.

### 2.2. Literature Management

Articles obtained from the three databases were managed by literature management software, NoteExpress2 (Aegean Software Co., Ltd., Beijing, China). The information (i.e., authors, title, journal, year, volume, and page) was extracted from each article and listed as a record. W. Zhang used NoteExpress2 to remove articles that were repetitively recorded in the three databases. Meanwhile, some articles that were considered not eligible for our study were excluded as well. The inclusion and exclusion criteria are shown in Table 1.

Although the repetitively recorded articles had been excluded, there still had been conditions leading to data overlap calculating; for example, one sample of patients could be reported in different aspects such as the aspect of treatment, or diagnosis, or nursing. Therefore, in order to avoid data overlap calculating, we classified the obtained articles into the following four types according to the contents: type I, articles about treatment and treatment-related studies; type II, articles about diagnosis and clinical evaluation; type III, articles about nursing and anesthesia; and type IV, articles about other aspects. The classification criteria are shown in Supplementary Table 1 in Supplementary Material available online at http://dx.doi.org/10.1155/2014/738548.

As sorting out these articles, we found that a large number of articles had been duplicately published in different journals. The prevalence of covert duplicate publications in Budd-Chiari syndrome articles in China had already been evaluated [24]. Thus, we defined covert duplicate publications as the criteria reported in that article. Two authors, W. Zhang and X. Qi, independently classified the articles according to our criteria and identified the covert duplicate publications according to the definition with the data extracted (see Section 2.3). In case of disagreement, a consensus was made through discussion between the two review authors. Duplicate publications were excluded from our study.

### 2.3. Data Extraction

It was noteworthy, in our study, that only the articles of type I were selected for the epidemiological data collection. This selection was mainly based on the following three reasons: first, the number of type I articles was the most in the four types of articles; second, the data our study needed were relatively complete in type I articles; and third, almost all patients treated had the diagnosis confirmed by surgery or venography.

Data extraction included the detailed information of all the type I articles, including authors, title, publication journal, year of publication, volume and issue, hospitals in which the cases were reported, provinces, cities, periods of enrollment (time of the study start and end), total number of cases reported (divided into three parts: number of initially treated primary BCS cases, number of retreated primary BCS cases, and number of secondary BCS cases), sex data, age range, mean age, duration, and the flag of duplicate publication (considered by the criteria of duplicate publications mentioned above). This procedure was completed by W. Zhang and X. Qi, and the extracted data were filed into an Excel table (Microsoft Office Excel 2007, Microsoft Corp., Redmond, Wash).

Not all type I articles were used as the origin of data extraction, and the eligible criteria included two major requirements: first, articles in which the initially treated primary BCS patients were reported and, second, articles in which the cases were reported from one center. Both requirements must be met. A few articles which reported both primary and secondary BCS patients or both initially treated and retreated patients were included regarding the number of the secondary BCS patients or the retreated patients which was small. Primary BCS is defined as an intravascular obstruction caused by thrombosis or membranous lesion of the hepatic venous outflow tract, while secondary BCS is referred to as the blockage caused by a tumoral invasion or compression.

### 2.4. Statistical Analysis

The number of BCS patients was calculated in every province. For each province, the number of BCS patients was calculated by a sum of cases reported from all hospitals in the cities of this province. In each hospital, the number of cases was counted by the end of five setting publication years (1990, 2000, 2005, 2010, and 2013). When one hospital had several studies of BCS patients treated in different enrollment periods, the number of cases was calculated as a total of the patients reported in each nonoverlap enrollment period aiming to obtain the maximum count. The number of the population of each province was acquired via the website of National Bureau of Statistics of the People's Republic of China.

## 3. Results

### 3.1. Literature Search and Management

**Figure 1:**
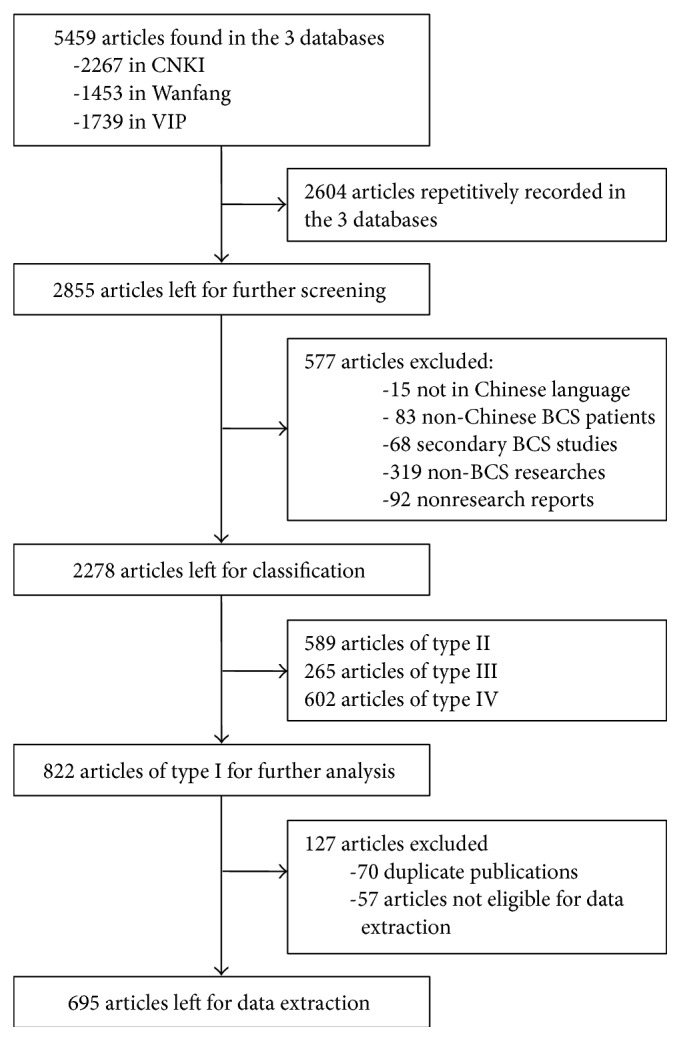
Flow chart for the literature management and selection. Twenty articles which were falsely recorded in the three databases (3 in CNKI, 4 in Wanfang, and 13 in VIP) were not included. BCS, Budd-Chiari syndrome; CNKI, China National Knowledge Infrastructure; VIP, Chinese Scientific and Technological Journal.

A total of 5459 articles were found in the three Chinese language databases, including 2267 in CNKI database, 1453 in Wanfang database, and 1739 in VIP database. Among them, 2604 articles repetitively recorded were excluded, and there were 2855 articles left. According to the inclusion and exclusion criteria, 2278 articles were eligible for the further classification. 822 articles were classified as type I articles, among which 70 were duplicately published and 57 were not eligible for data extraction, and finally 695 were eligible for data extraction. Literature management and selection was shown in the flow chart (Figure 1).

### 3.2. Demographics of BCS Patients

By the end of 2013, a total of 20191 BCS cases were reported (from 258 hospitals in 21 provinces, 4 autonomous regions, and 4 municipalities). Detailed data were obtained in 15692 cases, among which 15651 were primary BCS patients (including 115 retreated primary BCS patients) and 41 were secondary BCS patients; 9352 were males and 6286 females (sex data of 54 cases were not reported); the mean age was 36.29 ± 1.28 years (range of mean age: 33.90–47.20) and the range of age was 2–79 years; the duration of disease ranged from 1 day to 38 years and the mean duration was 48 months (Table 2).

### 3.3. Distribution of BCS Patients in China

**Figure 2:**
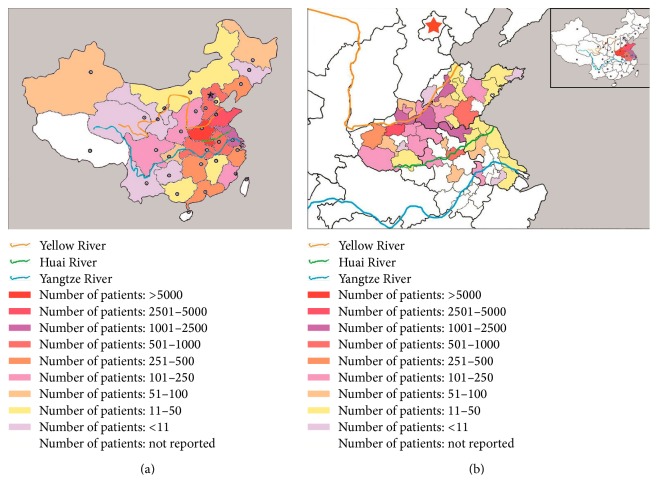
Distribution of BCS patients in China. (a) Distribution of BCS patients in 21 provinces, 4 autonomous regions, and 4 municipalities. (b) Distribution of BCS patients in the cities of Henan, Shandong, Jiangsu, and Anhui.

According to the cases reported from each province, autonomous region, and municipality, the distribution of BCS patients in China by the end of 2013 was presented in Figure 2(a). The top 5 high-prevalence areas which reported a large number of BCS patients were Henan, Shandong, Beijing, Jiangsu, and Anhui, and 80% of Chinese BCS patients were reported from these 4 provinces and 1 municipality. In a geographical view, except for Beijing, other 4 provinces are located in the downstream areas of Yellow River and the whole Huai River basin. In order to get a more detailed observation, we further analyzed the distribution of the cities in these 4 provinces (Figure 2(b)). We found that the cities lying in the area between Yellow River and Huai River had more BCS patients than the cities located in the either side of this area.

### 3.4. Epidemiological Estimation of BCS in China

For the epidemiological estimation, we calculated the total number of BCS patients reported by the end of 2010, 2005, 2000, and 1990, respectively (Table 3). We found, in recent 13 years, that the number of BCS patients was increased at a steady pace of approximately 1150 (830–1410) cases on average per year (from 5244 in 2000 to 20191 in 2013). Accordingly, at a conservative estimation, the incidence of BCS in China would be 0.88/million per year (the number of Chinese population was 1.3 billion by the end of 2010). Furthermore, we estimated the number of BCS patients who survived by the end of 2010 as follows. First, from 2000 to 2010, 10700 new cases were reported, the 10-year cumulative survival rate was 70% [23], and 7490 patients are estimated to be alive by the end of 2010; second, from 1990 to 2000, 4900 newly reported cases were found, 50% of these patients would be alive to the end of 2010 according to the long-term follow-up results of our center (not published), and other 2450 patients were added; finally the total number of BCS patients who survived by the end of 2010 was estimated to be approximately 10000 (9940), and accordingly the prevalence of BCS in China was estimated to be 7.69/million. In the same way, we calculated the incidence and prevalence of BCS in the provinces of Henan, Shandong, Jiangsu, and Anhui, respectively (Table 4). These four provinces, which had about a quarter of Chinese population, account for 70% of BCS patients in China. Although Beijing had a large number of BCS cases reported, we did not consider its high prevalence (52.35/million) or incidence (4.82/million per year) to be a true inflection of the really prevalent situation (see Section 4). Additionally, we calculated the incidence and prevalence of BCS in China without the top 5 high-prevalence areas, and the results were 0.28/million per year and 2.21/million, respectively.

## 4. Discussion

The present study reveals that a typical BCS patient in China is a man aged 30–40 with a membranous obstruction of the IVC and a relatively long duration of the disease, whereas in Western countries the most affected patients are young females with acute hepatic vein thrombosis [19]. In contrast to the previous studies which demonstrated either sex was predominantly affected in Asia [4, 6, 7, 16], our study reveals that the male patients are more common in China. Furthermore, this finding is also different from the reports from Western countries where the majority of BCS patients are females. Although recent data from a European cohort indicated a change in demographics as the male to female ratio was closer to 1 and median age was about 38 years [3], the demographics between the West and Asia were still different.

BCS is regarded as a rare disease in the Western countries. But when we refer to the “rare disease,” the definition is obscure. No single cutoff number has been agreed upon for which a disease is considered rare. A disease may be considered rare in one part of the world, or in a particular group of people, but may be still common in another one. There are different definitions of “rare disease” in different countries. In USA, a disease is considered rare if it is believed to affect fewer than 200,000 Americans [25]. In Europe, a disease or disorder is defined as rare when it affects less than 1 in 2000 citizens [26]. In Japan, the legal definition of a rare disease is one that affects fewer than 50,000 patients in Japan [27]. There is no widely accepted definition of rare disease in China, and only a consensus made by an expert group has defined the rare disease as the prevalence is lower than 1 in 500,000 or the neonatal morbidity is less than 1 in 10,000 [28]. This study, to our knowledge, is the first study which estimates the incidence and prevalence of BCS in China of the whole country. According to the Chinese definition of rare disease, generally speaking, BCS is not a rare disease in China (prevalence 7.69/million is more than 1 in 500,000). However, if we excluded the top 5 high-prevalence areas, the prevalence in other areas is considered to be rare and the incidence (0.28/million per year) is comparable to the results reported in Japan (0.13/million per year), France (0.36/million per year), and Sweden (0.8/million per year, age-standardized incidence) [16–18]. We notice that the high-prevalence areas are located in the downstream areas of Yellow River and the whole Huai River basin, especially for the cities lying in between Yellow River and Huai River. It is suggested that there might be some special environmental factors or living and dietary habits participating in the pathogenesis of membranous obstruction of the IVC (i.e., high iodine drinking water, high strength of the rural work, and wheat bran diet) [29, 30].

In present study, Beijing had the highest incidence and prevalence of BCS in China; however, this result did not reflect the really prevalent situation of Beijing. As we know, Beijing is the center of China with the most advanced medical techniques, and patients all over China came here in order to obtain a better therapeutic opportunity. Thus, among the BCS patients reported in Beijing, we believed that the most were not the inhabitants of Beijing but the ones who had the most serious diseases which could not be managed by the local hospitals. For the true incidence and prevalence of BCS in Beijing, we estimated them to be equal to the average of the whole country. Most of BCS patients who went to Beijing seeking for a better treatment came from the areas of high prevalence. For this reason, the true prevalence of these areas might be underestimated to some extent.

The first case of BCS in China which we retrieved was reported from Shenyang in 1957 [31]. By the end of 1990, there were more than 300 BCS cases reported, and from then on more and more cases had been reported. From 1991 to 2000, about 5000 new cases were reported; from 2001 to 2010, more than 10000 new cases were reported; from 2011 to 2013, more than 4000 new cases were reported. Comparing the number of new cases reported from 1991 to 2000 with that from 2001 to 2010, we found that the latter was more than twice as many as the former. This could be explained by the following reasons. First, because of the development of more advanced diagnosis techniques and the improved awareness of BCS, more and more patients who had missed diagnosis or had been misdiagnosed before had been correctly diagnosed; and second, as the national medical insurance system is being established, more and more rural patients took advantage of rural cooperative medical service and went to hospitals for diagnosis and treatment. We found that, from 2000 to 2013, the newly reported cases were at a steady pace of approximately 1150 on average per year. Although this number might be underestimated, the steady pace of newly reported cases suggested that this number reflects the newly diagnosed patients per year recently.

It is noteworthy that the estimation of incidence and prevalence in our study is based on the number of patients reported in Chinese articles, and the English published articles were not included. However, the total number of BCS patients in China would not be underestimated because of the duplicate publications [23]. Additionally, the accuracy of the estimation of incidence and prevalence depends on the following two premises: first, all BCS patients went to hospitals and got certain modality of treatment; and second, all BCS patients treated in the hospitals had been reported. We admit that, even if these two premises could be met in a vase extent, the total BCS patients might still be underestimated. The incidence and prevalence calculated based on literature survey should be viewed as a rough estimation which could be a reference to future epidemiological studies. Furthermore, a nationwide multicenter cooperative study on epidemiology and etiology in China is urgently needed to evaluate the prevalent conditions and find out the factors related to the formation of IVC membranous obstruction.

We acknowledge that there were several major limitations in our study. First, for the design of our study, it was not an epidemiological investigation, and the method used in present study was also unusual. As a rare disease, Budd-Chiari syndrome was not feasible to perform a common nationwide epidemiological investigation. Therefore, we use an alternative way to conduct an epidemiological estimation by literature survey, and there were apparently major drawbacks of the inaccurate estimation. We should emphasize that the epidemiological results estimated in our study were what we preferred to be viewed as a gross aspect of the general prevalence and distribution of BCS in China rather than a precisely calculated number from a well-designed epidemiological investigation. Although it was impossible to get a precise estimation through our method, we just tried to find a closer result to the real condition, which could delineate an overall impression of the whole Chinese patients with Budd-Chiari syndrome and could be a reference comparing with the results of Western countries, as well. Second, in order to collect the maximum number of cases reported in China, the qualities of the original data extracted from different studies were heterogeneous, and there was a conflict between the use of high-quality origin data and the collection of the maximum number of cases. Therefore, some information (such as sex data and mean age) was missing in a few articles, and some information (such as the type of BCS and the areas from where the BCS patients come) was unavailable in most articles, which precluded a further subgroup analysis. Third, some potential bias of the present study, including bias of underreporting cases and the cases of misdiagnosis and missed diagnosis, was unavoidable. Although we argued that the premises needed for the estimation could be satisfied under certain conditions, we admitted that our results should be viewed as a gross estimation other than an exact figure and should be interpreted cautiously.

## 5. Conclusion

Our study demonstrates that BCS is more prevalent in males and middle aged people in China. The distribution of Chinese BCS patients exhibits a heterogeneously geographic characteristic, and 70% patients are from the provinces of Henan, Shandong, Jiangsu, and Anhui, which are located in the downstream areas of Yellow River and the whole Huai River basin. With the top 5 high-prevalence areas taken into account, the overall incidence and prevalence of BCS in China are higher than those of the West, but if without them, the incidence and prevalence are comparable to the West.

## Supplementary Material

The articles were classified into four types mainly based on the contents concerning the most common subjects in clinical studies. Among these articles, the number of type I (treatment and treatment-related studies) was the most in all these four types. In order to obtain the maximum information, we select articles of type I as the origin of epidemiological data extraction. Meanwhile, this selection also could furthest avoid data overlap calculating. For the articles of type II (diagnosis and clinical evaluation) and type III (nursing and anesthesia), epidemiological data were lack in some studies. The articles of type IV mainly included basic studies and review articles.

## Figures and Tables

**Table 1:** Inclusion and exclusion criteria of the articles searched.

Inclusion criteria	Exclusion criteria
(1) Articles published in Chinese language on Chinese BCS patients.	(1) Articles not published in Chinese language or on non-Chinese patients.

(2) Researches in which primary BCS patients were studied or in which the most cases were constituted of primary BCS.	(2) Researches of patients with secondary BCS or articles of non-BCS researches, including hepatic veno-occlusive disease (recently renamed as sinusoidal obstruction syndrome), portal vein thrombosis, portal hypertension, cirrhosis, and other irrelevant studies.

	(3) Other nonresearch articles, including news reports, editorials, notifications, biographies, conference abstracts, and continuing medical education quizzes.

BCS: Budd-Chiari syndrome.

**Table 2:** Distribution and characteristics of BCS patients in China (2013).

Province	Number of cases			Characteristics	
	Total^†^	Primary (retreated)^‡^	Secondary	Male/female (*N*)^§^	Mean age (range) (years)
Henan	6586	4947 (2)	4	2960/1991	36.2 (5–79)
Shandong	4635	4063 (6)	5	2434/1634	36.3 (2–76)
Beijing	1965	1743 (14)	3	1025/721	34.3 (2–72)
Jiangsu	1931	876 (18)	0	472/404	36.4 (11–78)
Anhui	982	982 (33)	0	614/368	37.8 (4–76)
Hebei	908	398 (3)	0	252/146	37.4 (3–78)
Hubei	761	543 (4)	0	292/251	35.3 (6–78)
Guangdong	343	340 (27)	3	215/128	33.9 (2–67)
Liaoning	340	267 (0)	5	178/94	37.7 (8–78)
Zhejiang	319	115 (0)	0	61/51	43.5 (19–67)
Hunan	287	283 (2)	4	155/132	37.4 (16–66)
Shaanxi	210	210 (0)	0	146/64	38.0 (11–70)
Sichuan	163	152 (0)	11	67/48	37.1 (15–79)
Shanghai	143	141 (0)	1	101/41	38.1 (18–75)
Shanxi	107	85 (0)	0	54/31	36.8 (6–74)
Fujian	106	106 (0)	0	66/40	39.0 (21–75)
Xinjiang	77	77 (4)	0	50/27	36.9 (17–62)
Chongqing	59	59 (0)	0	33/26	38.9 (16–65)
Heilongjiang	53	50 (0)	3	37/16	39.0 (15–65)
Jiangxi	49	49 (0)	0	33/16	40.8 (17–68)
Yunnan	45	45 (2)	0	25/20	42.0 (19–65)
Tianjin	33	31 (0)	2	26/5	35.2 (7–57)
Inner Mongolia	28	28 (0)	0	18/10	NA
Guangxi	19	19 (0)	0	8/10	35.6 (17–46)
Guizhou	10	10 (0)	0	6/4	45.1 (21–70)
Qinghai	9	9 (0)	0	6/3	34.0 (12–65)
Ningxia	9	9 (0)	0	7/2	NA
Jilin	8	8 (0)	0	6/2	47.2 (24–66)
Gansu	6	6 (0)	0	5/1	NA

BCS: Budd-Chiari syndrome; NA: not available.

^†^Total number is not equal to the sum of primary and secondary BCS cases in some provinces because the detailed information is not reported in some cases.

^‡^Retreated cases refer to the primary BCS patients retreated and the articles reported the sole retreated patients are excluded from our study. A few articles which reported both primary and secondary BCS patients or both initially treated and retreated patients were included regarding the number of the secondary BCS patients or the retreated patients which was small.

^§^Sex data of 54 cases were not reported.

**Table 3:** Total number of BCS cases reported by the end of 4 setting years.

Year	Number of provinces	Number of cases			Characteristics	
		Total^†^	Primary (retreated)^‡^	Secondary	Male/female (*N*)^§^	Mean age (range) (years)
2010	29	15963	13340 (94)	26	7956/5298	35.6 (2–79)
2005	29	11798	9718 (47)	24	5910/3820	35.4 (2–79)
2000	28	5244	4893 (44)	11	3025/1879	33.5 (2–79)
1990	12	317	316 (7)	1	216/101	31.9 (11–62)

BCS: Budd-Chiari syndrome.

^†^Total number is not equal to the sum of primary and secondary BCS cases because the detailed information is not reported in some cases.

^‡^Retreated cases refer to the primary BCS patients retreated and the articles reported that the sole retreated patients are excluded from our study. A few articles which reported both primary and secondary BCS patients or both initially treated and retreated patients were included regarding the number of the secondary BCS patients or the retreated patients which was small.

^§^Sex data were not reported in some cases.

**Table 4:** Incidence and prevalence of the provinces of Henan, Shandong, Jiangsu, and Anhui.

Province	Population (million)	Newly reported cases (per year)	Patients survived by the end of 2010 (*N*)	Incidence (per million per year)	Prevalence (per million)
Henan	93	350	3650	3.76	39.25
Shandong	93	260	2160	2.80	23.23
Jiangsu	76	130	810	1.71	10.66
Anhui	59	60	440	1.02	7.46
