# Supplementary material for: Budd-Chiari Syndrome in China: A Systematic Analysis of Epidemiological Features Based on the Chinese Literature Survey
Source: Gastroenterol Res Pract. 2015 Oct 4;2015:738548. doi: 10.1155/2015/738548 (PMC4609452; doi:10.1155/2015/738548)
Supplement: Supplementary file 1 — The articles were classified into four types mainly based on the contents concerning the most common subjects in clinical studies. Among these articles, the number of type I (treatment and treatment-related studies) was the most in all these four types. In order to obtain the maximum information, we select articles of type I as the origin of epidemiological data extraction. Meanwhile, this selection also could furthest avoid data overlap calculating. For the articles of type II (diagnosis and clinical evaluation) and type III (nursing and anesthesia), epidemiological data were lack in some studies. The articles of type IV mainly included basic studies and review articles. [file 738548.f1.docx]

Supplementary Table 1 Classification Criteria of Articles.

| Classification | Definition |
| --- | --- |
| Treatment and treatment-related | Original articles reported the treatment of BCS patients (i.e., medicine, surgery, interventional radiological treatment, liver transplantation, and traditional Chinese medicine) and articles which presented the clinical analysis of BCS patients and mainly dealt with the therapeutic strategy. |
| Diagnosis and clinical evaluation | Original articles reported the studies of diagnosis of BCS patients (i.e., Roentgen ray, computerized tomography, magnetic resonance imaging, ultrasonography, and radioisotope scanning) or the evaluation of laboratory results or the severity of disease, and articles which dealt with the clinical misdiagnosis, missed diagnosis, and differential diagnosis. |
| Nursing and anesthesia | Original articles reported the studies of nursing observation and experience, cooperation in the surgery, and surgical and anesthetic care. |
| Others | Articles which could not be included into the above three types. |

BCS, Budd-Chiari syndrome.
